# Time-resolved vibrational spectroscopic study of molecular nanoaggregate photocatalysts†

**DOI:** 10.1039/d4sc03825h

**Published:** 2024-09-13

**Authors:** Chao Li, Tao Liu, Owen Thwaites, Adrian M. Gardner, Igor V. Sazanovich, Haofan Yang, Xiaobo Li, Andrew I. Cooper, Alexander J. Cowan

**Affiliations:** a Stephenson Institute for Renewable Energy and Department of Chemistry, University of Liverpool L69 7ZF UK acowan@liverpool.ac.uk; b Leverhulme Research Centre for Functional Materials Design, Materials Innovation Factory and Department of Chemistry, University of Liverpool Liverpool L7 3NY UK; c Stephenson Institute of Renewable Energy and Department of Physics, University of Liverpool Liverpool L69 7ZF UK; d Central Laser Facility, Research Complex at Harwell, STFC Rutherford Appleton Laboratory, Harwell Campus Didcot Oxfordshire OX11 0QX UK; e Materials Innovation Factory and Department of Chemistry, University of Liverpool Liverpool L7 3NY UK

## Abstract

The controlled aggregation of organic chromophores into supramolecular structures offers a way to control and tune photocatalytic activity. However, the underlying mechanisms of charge transfer and accumulation are still unclear. Time-resolved vibrational spectroscopy is a powerful structural probe for studying photogenerated intermediates. Here, we employ time-resolved infrared (TRIR) spectroscopy to study CNP (2,6-bis(4-cyanophenyl)-4-(9-phenyl-9*H*-carbazol-3-yl)pyridine-3,5-dicarbonitrile) and its supramolecular aggregates. We show that excitation of the charge transfer (CT) band of semi-crystalline nanofibers (CNP-f) gives rise to long-lived delocalised polarons, which form within the instrument response timescale. By contrast the CNP nanospheres (CNP-s) give rise to a shorter lived polaron that appears to have a greater degree of localization. CNP-f and CNP-s are known to show markedly different levels of photocatalytic activity for hydrogen and hydrogen peroxide formation which are rationalised owing to these differences in photodynamics immediately following photon absorption.

## Abstract 2

The controlled aggregation of organic chromophores into supramolecular structures offers a way to control and tune photocatalytic activity.

## Graphical abstract

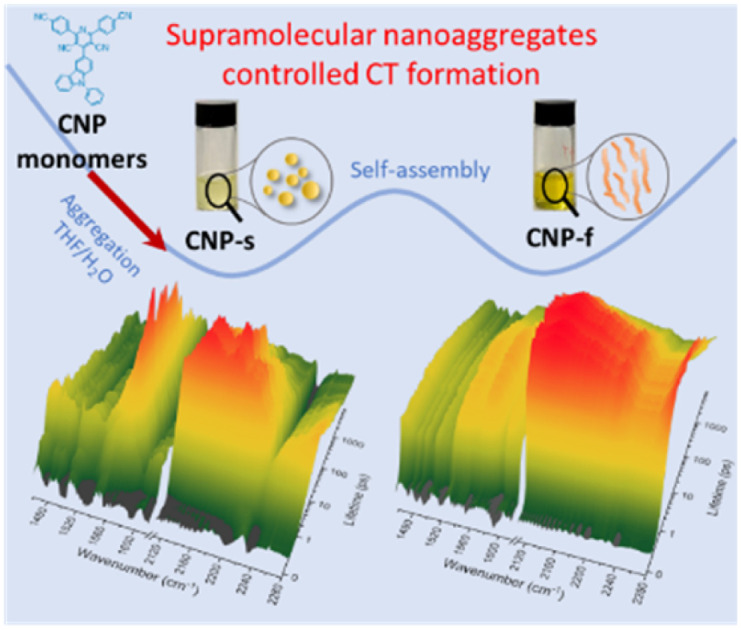

## Introduction

Photocatalysts absorb solar energy and use this to drive the formation of new chemical products, such as high energy density fuels. Organic photocatalysts for solar fuel production offer several advantages such as readily tunable optical gaps, synthetic control over structure, good processability, and preparation from earth-abundant materials.^1,2^ Beside the well-developed covalent organic polymers, such as covalent triazine-based frameworks (CTFs),^3–5^ covalent organic frameworks (COFs),^6–8^ linear conjugated polymers^9,10^ and conjugated microporous polymers (CMPs),^11,12^ noncovalent supramolecular assemblies based on small organic molecules have attracted recent interest.^13,14^

The large excitonic binding energy in organic photocatalysts is a barrier to the required efficient charge-separation; this can be improved with the introduction of charge transfer (CT) character within the excited electronic states, such as through incorporation of donor–acceptor (D–A) molecules.^15,16^ The mobility of excitonic and polaronic species over extended distances is known to be an important factor in organic(polymer) photocatalysts, for example, transport of excitons to sites of charge separation (such as the polymer/solvent interface) and the migration of separated charges to the sites of catalysis is required.^1,17^ Likewise, the degree of excitonic and polaronic localization can have a profound impact on photocatalytic activity with increased delocalization reducing the binding energy, facilitating charge separation.^18,19^ Both charge mobility and delocalization across extended distances can be enhanced by non-covalent interactions, such as π–π interactions between adjacent molecules within supramolecular aggregates,^20–22^ with the strength of the intermolecular interactions dependent on the distance and orientation of the sub-units.^18,19^ Therefore, as interaggregate distances and orientation are altered, the photophysics and subsequent photocatalytic activity can be modified. Reports are beginning to appear of changes in photocatalytic activity as a result of the self-assembly process.^18,23,24^ In principle, self-assembly pathways can be controlled by the choice of solvent, the concentration of the assembly unit, pH, and temperature.^25–27^ It follows that it should be possible to generate a range of distinct photocatalysts from a single assembly sub-unit. However, predicting (or even post-rationalising) how structure/assembly modifies photocatalytic activity is still challenging. It is therefore important that mechanistic studies are carried out to understand how assembly can control activity.

**Fig. 1:**
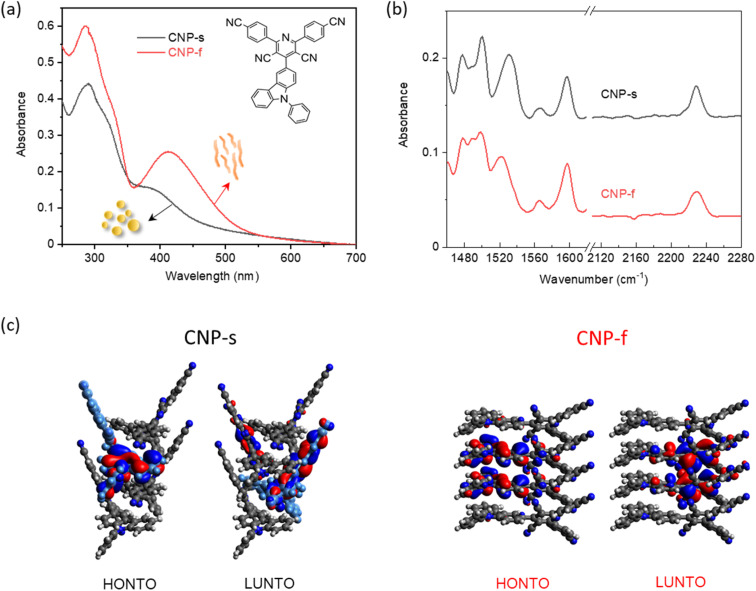
(a) UV/Vis absorption spectra of CNP-s and CNP-f in water (0.01 mg mL^−1^, pH = 7) measured in a 2 mm pathlength cuvette; insert is the chemical structure of CNP molecule (b) FTIR-ATR spectra of CNP-s and CNP-f aggregates in the solid form; (c) electron density distributions in the highest occupied natural transition orbitals (HONTO) and lowest unoccupied natural transition orbitals (LUNTO) of four-molecule-layers extracted from CNP-s (black) and CNP-f (red) by using time-dependent DFT in Gaussian 16 at the CAM-B3LYP/6-31G. Panel (c) is adapted with permission from ref. 18.

Time-resolved spectroscopies offer means to study the photophysics of photocatalysts. We have recently reported a simple donor–acceptor molecule CNP (2,6-bis(4-cyanophenyl)-4-(9-phenyl-9*H*-carbazol-3-yl)pyridine-3,5-dicarbonitrile, Fig. 1a) that can form distinct nanoaggregates (amorphous nanospheres, CNP-s or ordered nanofibres, CNP-f) depending on the solvent composition, each with contrasting photocatalytic activity.^18^ The CNP-f structures show a high quantum yield for photocatalytic hydrogen production in the presence of a Pt co-catalyst, whilst the CNP-s structures are largely inactive for hydrogen production. Conversely CNP-s is able to produce H_2_O_2_ efficiently through photocatalytic O_2_ reduction, whilst CNP-f is inactive. In the previous study, UV/Vis transient absorption (TA) spectroscopy was used to investigate the photophysical properties and the switch photocatalytic mechanism of CNP aggregates. TA spectroscopy showed the formation of a CT electronic state upon excitation of the CNP molecule in solution. The CNP-s nanoaggregates also showed a TA spectrum similar to the molecular CNP. In contrast, the TA spectrum of CNP-f showed distinctive excited state absorption bands which were tentatively assigned to the formation of a delocalised state across multiple units of the aggregate. However, a challenge is that UV/Vis spectra of the transient species are broad and often overlapped, making it difficult to assign spectral features to specific species, hindering the understanding of the photophysical mechanism which give rise to the differing photocatalytic activities of the different nanoaggregates.

Time-resolved vibrational spectroscopies (Raman and infrared) are able to directly report on the chemical structure of short-lived intermediates.^28,29^ Time-resolved infrared spectroscopy (TRIR) has been used to examine the photoinduced charge transfer processes and solar fuel conversion with studies of molecular photocatalysts and inorganic materials reported.^30–33^ A smaller number of TRIR studies of organic photocatalysts exist, primarily studying carbon nitride-like materials.^34,35^ TRIR spectroscopy is particularly useful for studying the nature and formation of polaronic states in organic photocatalysts. In TRIR studies on related organic photovoltaic systems, long-lived charges have been reported to accumulate across π-stacked structures to form delocalised polarons (electron polarons, hole polarons or polaron pairs) and may be directly observed through broad electronic absorption features that extend into the mid infrared (mid-IR) spectral region,^36–38^ and narrow vibrational resonances assignable to the transient polaronic species. Polaronic species can also be indirectly observed in TRIR studies owing to inducing perturbations in the geometric and electronic structure within neighbouring substituents, resulting in previously IR dark modes becoming IR active, commonly denoted as infrared activated vibrations.^36,38,39^ Therefore, analysis of the mid-IR spectral region can give information on both the yield and degree of localisation of photogenerated charge separated states. Here, we employ TRIR spectroscopy as a structural probe to gain the insight into the interplay of molecular packing mode in controlling the nature of the transient species formed in different CNP aggregates, in the absence of the Pt co-catalyst, to determine how the aggregation controls photophysical and hence photocatalytic properties.

## Results and discussion

The structure of the CNP molecule and the UV/Vis absorption spectra of the two different CNP aggregates are shown in Fig. 1a. We showed previously that the lowest energy, broad transition, extending from UVA into the visible region (350–400 nm) is assignable to a CT state formed between the pyridinedicarbonitrile acceptor unit and a carbazole donor unit (Fig. 1a). The CT band of CNP-f (412 nm, 3.01 eV) is red-shifted relative to that of CNP-s (380 nm, 3.26 eV Fig. 1a).^18^ We also detailed the structure of these aggregates.^18^ Here we focus on the vibrational spectra of the materials, but it is important to briefly reiterate the most pertinent points regarding the structure. CNP-s has a morphology of spheres of ∼100 nm diameter and XRD indicates that these spheres are amorphous. CNP-f fibres have a width ∼40 nm and show peaks in the XRD pattern indicating a degree of structural order with a *d* spacing of 3.7–3.9 Å, in-line with the structure of a single crystal of CNP that was grown showing strong π stacking along the fibre axis.^18^

The ground state Fourier transform infrared (FTIR) spectrum (1460–1620 and 2110–2280 cm^−1^) of CNP in solution (CHCl_3_) shows multiple peaks at 1473, 1489, 1497, 1507, 1521, 1534, 1540, 1559, 1569, 1576, 1599 1608 and 2230 cm^−1^ (Fig. S1†). These bands are assigned to ring/carbon backbone modes except for 2230 cm^−1^, which is assigned to *ν*(C≡N) modes of the acceptor subunit, Table S1.† In CNP-s the FTIR peaks in the region of 1460–1620 cm^−1^, observed at 1478, 1488, 1500, 1531, 1566 and 1597 cm^−1^ (Fig. 1b and S2†), are broader than those of the CNP molecule in solution, due to π–π stacking interactions between the aromatic rings in nanoaggregates.^40^ The FTIR spectrum of CNP-f shows similar features to CNP-s in the 1460–1620 cm^−1^ range; however, notably, for the C≡N mode the band broadens and slightly shifts to higher wavenumber, in both the powder form and in aqueous (D_2_O) suspension (Fig. S3†).

**Fig. 2:**
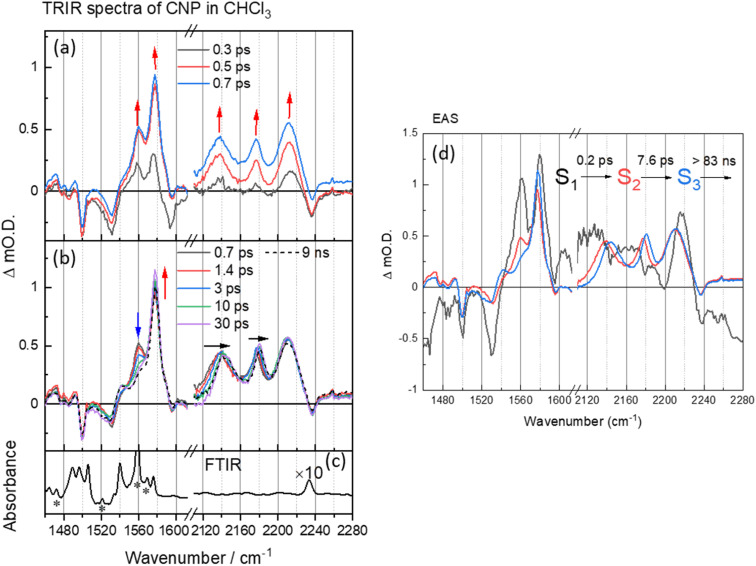
(a and b) ps–ns time-resolved infrared (TRIR) spectra of CNP in CHCl_3_ (100 μM) following excitation at 380 nm, shown at different time delays. The arrows show the direction of the spectral evolution with time. (c) The ground-state FTIR spectrum of CNP in CHCl_3_ (100 μM). (d) The corresponding evolution associated spectra (EAS) and time constants generated through 3 compartment sequential pathway global lifetime analysis (GLA) of data in part (a). In (b), the FTIR spectrum in CN region (black line) has been multiplied by a factor of 10 for clarity. The asterisks refer to bands from the CHCl_3_ solvent.

Initially TRIR spectra of CNP in solution (CHCl_3_) following CT band excitation (380 nm) were recorded across two spectral regions corresponding to those of the ring modes/*ν*(C=C) (1460–1620 cm^−1^) and *ν*(C≡N) (2110–2240 cm^−1^), Fig. 2. At the earliest times studied after excitation the ground state *ν*(C≡N) band (2237 cm^−1^) is observed as a negative feature, which we assign as a ground state bleach and we measure three new bands at 2137, 2176 and 2212 cm^−1^. These new transient bands shift in wavenumber over the first ∼30 ps, and at 50 ps are appear at 2144, 2180, 2212 cm^−1^. DFT calculations of the IR spectrum of the ground and CT state of the CNP molecule reproduce the trends observed in the experimental data (Fig. S5, S6 and Table S1†). Notably, three *ν*(C≡N) modes are present in the experimental TRIR spectrum, which is reproduced in the calculated spectrum of the CT state of CNP due to the lifting of the (close to) degeneracy of the *ν*(C≡N) modes in the ground state.^41–43^ The *ν*(C≡N) modes in the excited state occur at lower wavenumbers than the ground state owing to increased electron density at the acceptor (pyridinedicarbonitrile) unit, as also observed in the calculated spectrum of the CT state. Hence, we conclude that the initially formed exciton has charge-transfer character, confirming the assignment of the 386 nm band observed in the ground-state UV/Vis spectrum to a transition to a CT excited electronic state (Fig. S7†).

Multiple *ν*(C=C) bands are well resolved in the TRIR spectrum, with the bleaching peaks minima at 1490, 1530, 1575 and 1595 cm^−1^, corresponding to the C=C aromatic vibration in the pyridine and carbazole skeleton^44,45^ and three new transient *ν*(C=C) bands observed instantaneously upon excitation at 1540, 1560 and 1577 cm^−1^, consistent with changes in this spectral region predicted from the DFT calculations (Fig. S6 and Table S1†). In the first 30 ps after excitation we note a change in the relative intensity of the bands at 1560 and 1577 cm^−1^, however, unlike the transient *ν*(C≡N) bands, the transient bands observed in the 1490–1620 cm^−1^ show little evolution in wavenumber with time.

To explore the fast (ps–ns) processes occurring following excitation of CNP in solution we have carried out Global Lifetime Analysis (GLA) using a model consisting of three sequential components (see ESI† for full details). The initial process (S_1_ → S_2_) exhibits ultrafast time constant (∼0.2 ps), which is close to the instrument response time, and little can be inferred from the appearance of the corresponding evolution associated spectrum (EAS) of S1. The corresponding EAS of S_2_ shows the newly formed transient bands described above. The subsequent process (S_2_ → S_3_) with a lifetime of 7.6 ps shows that the intensity of the transient bands centred at 1577 and 1540 cm^−1^ assigned to ring modes on both the donor and acceptor units (Table S1†) increase whilst the band at 1560 cm^−1^ which is localised on the acceptor decays (Table S1 and Fig. S8†). Concomitant with this the three CN stretching vibrations initially located at 2212, 2176 and 2136 cm^−1^ shift to higher wavenumber on the same timescale (7.6 ps, Fig. S9†). Notably, TA UV/Vis spectroscopy of CNP in solution shows a blue-shift in the excited state absorption of the CNP molecule with a similar lifetime (Fig. S10†).^18^ We assign the spectral changes from S_2_ to S_3_ in EAS to be due to a combination of vibrational relaxation, solvent reorganization and relaxation of the CNP molecule, likely twisting around the linking C–C bond of the D–A structure of CNP.^18,46–48^ The slowest process (S_3_→) with a time constant of ∼83 ns is significantly beyond the longest-timescale probed in these experiments (9 ns) and it simply indicates that the CT state persists to timescales beyond that studied in the ps–ns TRIR study. To explore the fate of the CT state of CNP molecules in CHCl_3_ we have also carried out a separate TRIR study on the ns–μs TA timescale (Fig. S11†) that shows that decay to the ground state occurs with a lifetime of *ca.* 1 μs (Fig. S12 and Table S2† for the kinetic trace and detailed fits) with no further evolution of the transient spectrum occurring on the ns–μs timescale.

**Fig. 3:**
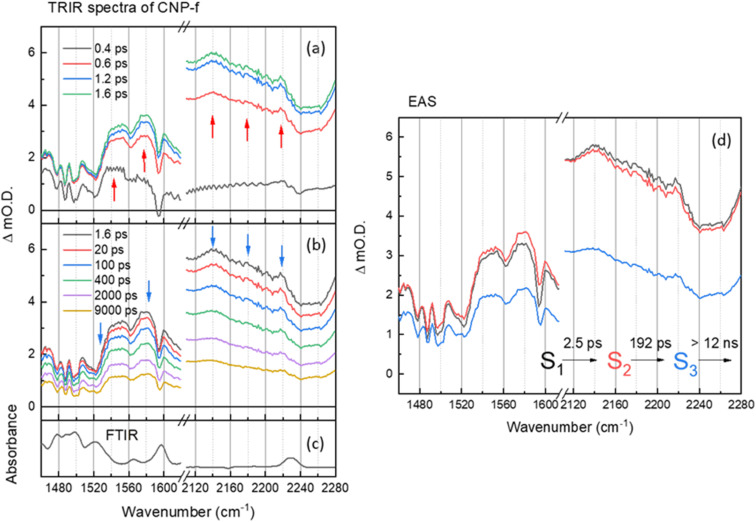
(a and b) ps–ns time-resolved infrared (TRIR) spectra of CNP-f films of the aromatic C=C and CN spectral regions following excitation at 380 nm, shown at different time delays. The arrows show the direction of the spectral evolution (c) the FTIR spectra CNP-f. (d) The corresponding evolution associated spectra (EAS) and time constants generated through 3 compartment sequential pathway global lifetime analysis (GLA) of data in part (a and b).

The TRIR and corresponding EAS spectra of the CNP-f film following 380 nm excitation show distinct differences to the TRIR data for the CNP in CHCl_3_ solution (Fig. 2 and 3). Within 0.8 ps of excitation of CNP-f a very broad transient absorption across the mid-IR region is observed (1460–2280 cm^−1^) and it increases in intensity for approximately ∼1.5 ps. Negative going vibrational modes are present at 1524, 1562, 1595 and 2239 cm^−1^ and we also observe new transient bands at 1554, 1577, 2140 and 2217 cm^−1^ (positions at 5 ps); however, the 2180 band observed for CNP in solution is not clearly observed. Through comparison with the experimental and computational spectra of CNP in solution, these transient bands are assignable to the stretching modes of the ring (1500–1600 cm^−1^) and cyano groups (2100–2250 cm^−1^) of the excited state. The newly formed (positive) transient IR bands at 2140 and 2217 cm^−1^ assigned to *ν*(C≡N) vibrations are again observed at lower wavenumber in the TRIR spectrum than observed in the ground state FTIR spectrum indicative of the transfer of charge from the donor to the acceptor of CNP, confirming that 380 nm excitation predominately populates the CT excited state in CNP-f. The broad transient absorption across the mid-IR is assigned to an electronic absorption of the polaronic state formed, which is electronic in nature, with similar broad mid-IR absorptions reported for organic semiconductors used for photovoltaics.^36,49,50^ However, it cannot be assigned definitively to a specific polaronic species *i.e.* polaron-pair or individual charged species. It is notable that the ground state (FTIR) *ν*(CN) band at 2230 cm^−1^ for CNP-f does not coincide with the centre of the broad negative going signal observed in the TRIR spectra (∼2250 cm^−1^, Fig. 3) and that there is poor agreement between the peak minima of the negative bands in the TRIR spectrum and the ground state FTIR generally for CNP-f. Similar observations have been reported in TRIR spectra of organic polymers, in which narrow vibrational transitions interact with the broad electronic transition give rise to Fano-type interference effects in the transient spectrum,^49,51^ which maybe trivially identified through apparent shifts of “bleach” signals compared to ground state vibrational wavenumbers.

Interestingly the resonant TRIR bands of CNP-f remain very broad for >1 ns upon excitation indicating that the breadth is not due to the formation of a vibrationally hot state. GLA of the TRIR spectra recorded on the ps–ns timescale shows the initial (S_1_) fast formation of the broad electronic absorption and the formation of broad vibrational modes. The relaxation process from S_1_ to S_2_ with a time constant of 2.5 ps shows slight increase in intensity of polaronic electronic absorption in the region of 1460–1620 cm^−1^, as observed by a shift in the broad baseline signal to more positive Δ*m*O.D. values, and a slight decrease in the intensity of the polaronic electronic absorption in the region of 2120–2280 cm^−1^, as observed by a shift in the baseline signal to less positive Δ*m*O.D. values, however the positions and widths of the *ν*(C≡N) vibrational bands are largely unchanged. The subsequent process (S_2_ → S_3_) exhibits no significant change in peak width (or wavenumber) of the vibrational modes. The final decay process (12 ns) is beyond the longest time delay in this measurement and again indicates that the CT persists to timescales beyond that studied. The requirement of multiple exponential functions, which have EAS with similar structure, to adequately fit the TRIR data is a result of the complex decay kinetics of the initially formed polaronic state.^52,53^ Consist with this, the broad widths of the vibrational bands observed in the TRIR spectra across the ps–ns time scale of CNP-f are indicative of either a diversity of polaron states being present (inhomogeneous broadening) or the presence of delocalised polaronic states,^52,53^ with linewidths of vibrational modes observed in transient IR spectroscopy correlated to the degree of excitonic delocalisation, being notably pronounced when the excited electronic state is delocalised over monomers which are bound through intermolecular interactions.^54^ This is consistent with previous DFT calculations which indicate the initially formed CT excitonic state is spread over multiple CNP subunits in a model nanofiber structure due to the close π–π stacking, which would be anticipated to facilitate delocalisation of the polaron across multiple CNP units within the aggregate. In such a system, owing to the close proximity of the charges within the HONTO and LUNTO, both like and counter charges will interact coulombically, influencing the initial local electronic structure of the polymer, and can be described as an exciton with charge transfer character.^18^

**Fig. 4:**
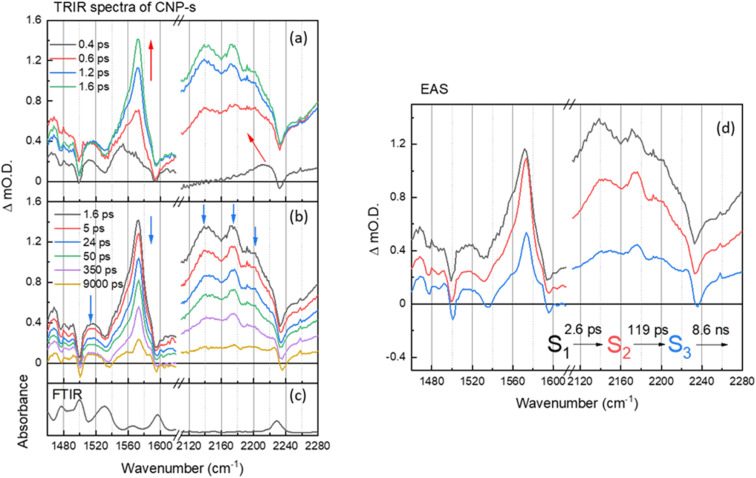
(a and b) ps–ns time-resolved infrared (TRIR) spectra of CNP-s films of the aromatic C=C and CN spectral regions following excitation at 380 nm, shown at different time delays. The arrows show the direction of the spectral evolution. (c) The FTIR spectrum of CNP-s. (d) The corresponding evolution associated spectra (EAS) and time constants generated through 3 compartment sequential pathway global lifetime analysis (GLA) of data in part (a and b).

On the ultrafast (ps–ns) timescale the TRIR and corresponding EAS spectra of the CNP-s aggregate film following excitation of the CT band is different again. Bleached ground state bands are observed (1476, 1500, 1535, 1595 and 2233 cm^−1^) and transient bands at 1468, 1480, 1490, 1515, 1554, 1572, 2140, 2173 and 2199 cm^−1^ are present in Fig. 4a and b. The trend in the ground state and transient *ν*(C≡N) vibrational wavenumbers are again indicative of CT from the carbazole donor unit to the pyridinedicarbonitrile acceptor unit having occurred. The vibrational bands of the CNP-s CT state become increasingly narrower and more resolved within the first 2 ps, indicative of initial vibrational cooling. The rapid formation of narrow, well defined vibrational bands is indicative that the excited state is significantly more localized than for CNP-f. Similar to the TRIR spectra of CNP-f on the ultrafast timescale the resonant TRIR modes of CNP-s are overlapped with broad, but much weaker absorption, across the mid-IR, which is again assigned to an electronic absorption of the polaronic species formed. Supporting the assignment is the broad negative going band between ∼2230 and ∼2260 cm^−1^, which does not correspond to the position of the much narrower ground state *ν*(CN) band, therefore, as in the TRIR spectrum of CNP-f, we conclude this feature arises from the interaction of the transient CN vibration with the electronic absorption.^36,38,55,56^

The EAS of initial formed state S_1_ from the GLA of CNP-s is assigned to the formation of the CT exciton, Fig. 4d. The relaxation process (S_1_ → S_2_) with a lifetime of 2.6 ps describes the narrowing of the transient band(s) between ∼1540 and ∼1580 cm^−1^, a decrease in intensity of the broad background assigned to the electronic transition of the polaron, and we also measure a shift in the *ν*(CN) frequency. The shift of *ν*(CN) frequency on the ps timescale of CNP-s is similar to the behaviour of CNP in solution where an initial cooling and conformational change (twisting) was proposed to occur with a lifetime of 7.6 ps. The CNP-s aggregate has decreased conformational freedom compared to the CNP molecule in solution but it is notable that a degree of relaxation is observed to occur with amorphous CNP-s and not with the semi-crystalline CNP-f aggregate and that relaxation leads to a narrowing of the vibrational bands and a decrease in intensity of the absorption assigned to the polaronic state. The subsequent process (S_2_ → S_3_) for 119 ps with an unchanged TRIR shape in vibrational modes shows the decay of broad background electronic absorption and transient vibrational absorptions, indicative of decay of the transient species to the ground state. As in the results of the GLA obtained for CNP-f as, we note that the requirement of multiple exponential functions, which have EAS with similar structure, to adequately fit the TRIR data is a result of the complex decay kinetics of the initially formed polaronic state. The broad distribution of decay kinetics observed in the fitting of the TRIR data of CNP-s indicate that there is a significant degree of disorder within the transient states populated, despite the well resolved vibrational bands observed in the TRIR spectra, consistent with the amorphous structure of CNP-s.

**Fig. 5:**
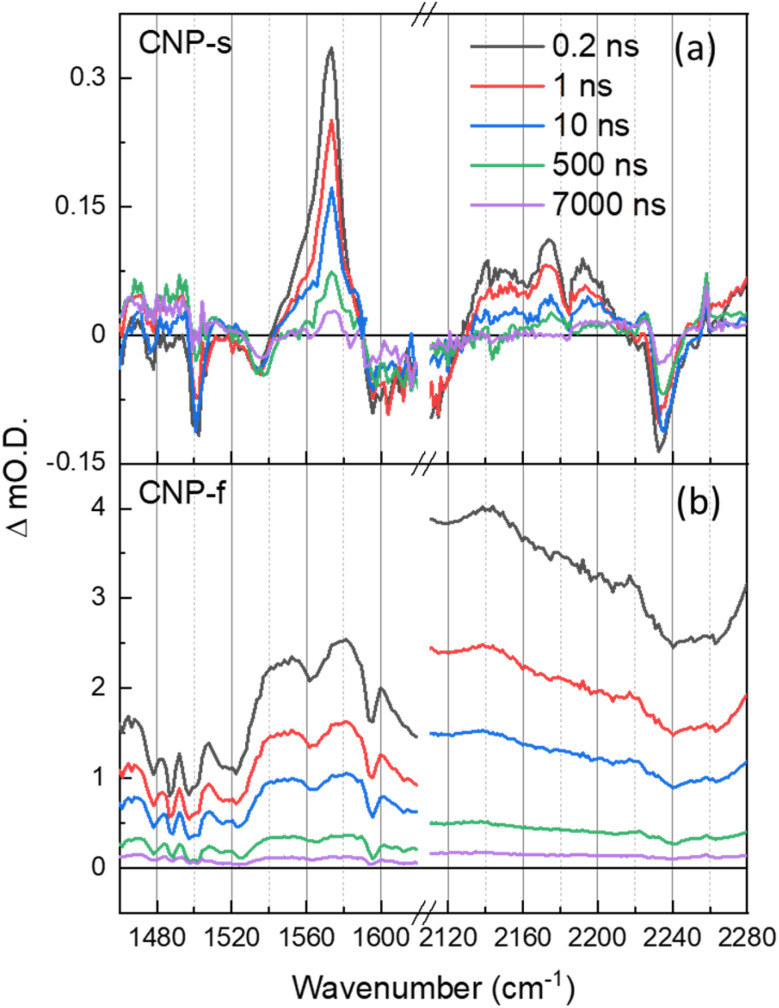
ns–μs TRIR spectra of CNP-s (a) and CNP-f (b) films in the aromatic C=C and CN stretch spectral regions following 380 nm excitation, shown at same time delays indicated.

The TRIR spectra of CNP-s and CNP-f recorded on the ns–μs timescale show that the broad electronic absorption feature rapidly decays with CNP-s and that by 10 ns only weak vibrational bands are present, Fig. 5. The narrower resonant TRIR bands of CNP-s coupled to the fast decay of the electronic absorption suggest a greater degree of localisation, hence increased binding energy, with the amorphous structure hindering charge delocalization. In contrast the CNP-f TRIR spectrum shows that the delocalised polaronic state is retained until >500 ns (Fig. 5b). Fitting of the decay traces for the TRIR bands in the slow (ns–μs) experiments that are assigned to the ring-mode and cyano groups of the excited state (CNP-s: 1571, 2140 cm^−1^; CNP-f: 1577, 2140 cm^−1^) to three exponential functions allows for comparison to the lifetime of the broad electronic background TRIR response (plotted for CNP-s and CNP-f: 2275 cm^−1^), Fig. S14.† The amplitude weighted average lifetime offers a simple way to assess the stability of the excited states present in the slow (ns–μs) TRIR study. The lifetime of the broad background of CNP-f assigned to electronic absorption of the polaronic state is 444 ± 108 ns. The lifetime of the resonant vibrational modes of the CNP-f aggregate is similar (523 ± 98 ns (1577 cm^−1^) and 414 ± 71 ns (2140 cm^−1^)). We conclude that the broad electronic signals observed across the mIR wavelengths probed, and the vibrational bands observed for CNP-f, arise from the same transient species. The broad electronic absorption in CNP-s is much weaker in the ps–ns timescale than in CNP-f, and is very weak in ns–μs spectra in Fig. 5, and is only present on a small number of delays measured preventing accurate determination of the lifetime, but it is clear that the lifetime of the electronic absorption of the polaronic state of CNP-s measured in the mIR wavenumbers probed is <10 ns, significantly less than CNP-f. These results are consistent with our previous μs–s UV-vis TA experiments in which a transient population persisted on the millisecond timescale for CNP-f, with minimal CNP-s excited state remaining by 10 μs.^18^ This observation indicates that there are stark differences in the electronic structure of the excited states of the two nanoaggregates. Interestingly vibrational modes of a CNP-s CT state do persist well after the broad background signal has decayed (*e.g.* 1571 cm^−1^, 590 ± 136 ns). This difference in lifetime is consistent with the fitting of the ps–ns TRIR data which indicated that that there is a significant degree of disorder within the transient states populated. The spectra recorded at the longest time delays of CNP-s (>10 ns) closely resemble that of the localised CT state of CNP in solution (Fig. S15†), suggestive that the longest lived states are considerably more localised, hence more similar to the isolated CNP molecule, than CNP-f (Fig. 1c and d).

CNP-f is an active photocatalyst for H_2_ evolution in the presence of a Pt co-catalyst, whilst CNP-s is not. TA (UV/Vis) spectroscopy demonstrates that electron transfer to Pt happens on the ps timescale with CNP-f, but that electron transfer does not occur from CNP-s to Pt.^18,57^ Although shorter lived the polaronic state of CNP-s is initially formed and a (small) population does persist for >500 ps (Fig. S14a†) demonstrating that lifetime is not the sole reason for the lack of electron transfer to the Pt co-catalyst and subsequent H_2_ evolution. Instead it is proposed that the formation of a delocalized polaron following excitation of the CT band of the semi-crystalline CNP-f is key in enabling charge transport throughout the polymer. This enhances electron transfer to Pt required for subsequent H_2_ formation, and hole transport to the sacrificial electron donor (ascorbic acid) which we have previously shown is vital to prevent hole accumulation within the polymer.^18^ Distribution of charge over a greater number of atoms may be beneficial for a number of reasons. First, binding energies will be decreased, facilitating charge transfer to the catalyst and sacrificial regents.^58,59^ Second, delocalization leads to increased mobility of charges facilitating transport through and across aggregate structures which will be critical as Pt catalysts are present as nanoparticles within the material.^60,61^ We can also hypothesize as to why CNP-s is active for H_2_O_2_ production (*via* O_2_ reduction), whilst CNP-f is not. In this case it seems likely that the catalyst free O_2_ reduction is favored with the localized polaronic state of CNP-s.

## Conclusions

The TRIR data supported by DFT calculations provides clear evidence that following excitation of the CT band there is charge transfer from the carbazole donor unit to the pyridinedicarbonitrile acceptor regardless of the aggregation state of the CNP. For CNP in solution the initially formed CT state undergoes rapid initial relaxation with a lifetime of *ca.* 8 ps, which is proposed to be a combination process of vibrational cooling, solvent reorientation and relaxation of the CNP molecule due to conformation freedom in solution. For CNP-s evidence of rapid polaron formation is present but it is notable that the state is short lived (<10 ns) and the well-defined TRIR bands suggest a degree of localization/limited distribution of charge accumulation sites, suggesting less efficient electron transfer across the aggregate structure to co-catalyst Pt to drive H_2_ evolution. Conversely the catalyst free O_2_ reduction is favored with the localized polaronic state of CNP-s. For CNP-f the TRIR spectra, along with our previously reported DFT calculations, supports the formation of a delocalized polaron following excitation of the CT band that is significantly longer lived than for CNP-s. Long-lived delocalized polarons and the ordered semi-crystalline structure lead to enhanced mobility of charges facilitating transport through and across aggregate structures to the final H_2_ production site on co-catalyst Pt nanoparticles. This work highlights the importance of polaron formation in different molecular packing modes for organic photocatalytic applications and provides insights into the structure–function relationship of molecular nanoparticle photocatalysts.

## Data availability

Underpinning time resolved spectra are freely available on the University of Liverpool Research Data Catalogue (https://doi.org/10.17638/datacat.liverpool.ac.uk/2804).

## Author contributions

The experiments were performed by C. L., and T. L. performed DFT calculations. A. I. C. and A. J. C. supervised and designed the project. The paper was written by C. L., A. M. G. and A. J. C. All authors contributed to data analysis, interpreted the data, and approved the final manuscript.

## Conflicts of interest

The authors declare no competing financial interests.

## Supplementary Material

SC-OLF-D4SC03825H-s001

## References

[cit1] Li C., Cowan A. J., Gardner A. M. (2022). Chem. Phys. Rev..

[cit2] Bai Y., Hippalgaonkar K., Sprick R. S. (2021). J. Mater. Chem. A.

[cit3] Kong D., Han X., Shevlin S. A., Windle C., Warner J. H., Guo Z. X., Tang J. (2020). ACS Appl. Energy Mater..

[cit4] Lan Z., Fang Y., Zhang Y., Wang X. (2018). Angew. Chem., Int. Ed..

[cit5] Kong D., Han X., Xie J., Ruan Q., Windle C. D., Gadipelli S., Shen K., Bai Z., Guo Z., Tang J. (2019). ACS Catal..

[cit6] Fu Z., Wang X., Gardner A. M., Wang X., Chong S. Y., Neri G., Cowan A. J., Liu L., Li X., Vogel A., Clowes R., Bilton M., Chen L., Sprick R. S., Cooper A. I. (2020). Chem. Sci..

[cit7] Xu J., Yang C., Bi S., Wang W., He Y., Wu D., Liang Q., Wang X., Zhang F. (2020). Angew. Chem., Int. Ed..

[cit8] Zang Y., Wang R., Shao P. P., Feng X., Wang S., Zang S. Q., Mak T. C. W. (2020). J. Mater. Chem. A.

[cit9] Fu Z., Vogel A., Zwijnenburg M. A., Cooper A. I., Sprick R. S. (2021). J. Mater. Chem. A.

[cit10] Bai Y., Li C., Liu L., Yamaguchi Y., Bahri M., Yang H., Gardner A., Zwijnenburg M. A., Browning N. D., Cowan A. J., Kudo A., Cooper A. I., Sprick R. S. (2022). Angew. Chem., Int. Ed..

[cit11] Sprick R. S., Bai Y., Guilbert A. A. Y., Zbiri M., Aitchison C. M., Wilbraham L., Yan Y., Woods D. J., Zwijnenburg M. A., Cooper A. I. (2019). Chem. Mater..

[cit12] Wang Z., Yang X., Yang T., Zhao Y., Wang F., Chen Y., Zeng J. H., Yan C., Huang F., Jiang J. X. (2018). ACS Catal..

[cit13] McDowall D., Greeves B. J., Clowes R., McAulay K., Fuentes-Caparrós A. M., Thomson L., Khunti N., Cowieson N., Nolan M. C., Wallace M., Cooper A. I., Draper E. R., Cowan A. J., Adams D. J. (2020). Adv. Energy Mater..

[cit14] Smith C. L., Mears L. L. E., Greeves B. J., Draper E. R., Doutch J., Adams D. J., Cowan A. J. (2019). Phys. Chem. Chem. Phys..

[cit15] Yu J., Chang S., Xu X., He X., Zhang C. (2020). ACS Sustain. Chem. Eng..

[cit16] Yang J., Jing J., Zhu Y. (2021). Adv. Mater..

[cit17] Banerjee T., Podjaski F., Kröger J., Biswal B. P., Lotsch B. V. (2020). Nat. Rev. Mater..

[cit18] Yang H., Li C., Liu T., Fellowes T., Chong S. Y., Catalano L., Bahri M., Zhang W., Xu Y., Liu L., Zhao W., Gardner A. M., Clowes R., Browning N. D., Li X., Cowan A. J., Cooper A. I. (2023). Nat. Nanotechnol..

[cit19] Lee J., Luke J., Ahn H., Kim D., Jin C., Kim M. H., Won Y. S., Yoon M., Kim J. (2022). Adv. Electron. Mater..

[cit20] Weingarten A. S., Kazantsev R. V., Palmer L. C., Fairfield D. J., Koltonow A. R., Stupp S. I. (2015). J. Am. Chem. Soc..

[cit21] Cyran J. D., Krummel A. T. (2015). J. Chem. Phys..

[cit22] Li Q., Li Z. (2020). Acc. Chem. Res..

[cit23] Lu J., Li Z., An W., Liu L., Cui W. (2019). Nanomaterials.

[cit24] Kazantsev R. V., Dannenhoffer A. J., Weingarten A. S., Phelan B. T., Harutyunyan B., Aytun T., Narayanan A., Fairfield D. J., Boekhoven J., Sai H., Senesi A., O'Dogherty P. I., Palmer L. C., Bedzyk M. J., Wasielewski M. R., Stupp S. I. (2017). J. Am. Chem. Soc..

[cit25] Wang S. P., Lin W., Wang X., Cen T. Y., Xie H., Huang J., Zhu B. Y., Zhang Z., Song A., Hao J., Wu J., Li S. (2019). Nat. Commun..

[cit26] Wang Y., Xu H., Zhang X. (2009). Adv. Mater..

[cit27] Wang C., Wang Z., Zhang X. (2012). Acc. Chem. Res..

[cit28] Lewis-Borrell L., Sneha M., Clark I. P., Fasano V., Noble A., Aggarwal V. K., Orr-Ewing A. J. (2021). J. Am. Chem. Soc..

[cit29] Piercy V. L., Saeed K. H., Prentice A. W., Neri G., Li C., Gardner A. M., Bai Y., Sprick R. S., Sazanovich I. V., Cooper A. I., Rosseinsky M. J., Zwijnenburg M. A., Cowan A. J. (2021). J. Phys. Chem. Lett..

[cit30] Munson K. T., Kennehan E. R., Asbury J. B. (2019). J. Mater. Chem. C.

[cit31] Brown A. M., McCusker C. E., Carey M. C., Blanco-Rodríguez A. M., Towrie M., Clark I. P., Vlček A., McCusker J. K. (2018). J. Phys. Chem. A.

[cit32] Huang J., Gatty M. G., Xu B., Pati P. B., Etman A. S., Tian L., Sun J., Hammarström L., Tian H. (2018). Dalton Trans..

[cit33] Cerpentier F. J. R., Karlsson J., Lalrempuia R., Brandon M. P., Sazanovich I. V., Greetham G. M., Gibson E. A., Pryce M. T. (2021). Front. Chem..

[cit34] Ben-Refael A., Benisti I., Paz Y. (2020). Catal. Today.

[cit35] Benisti I., Shaik F., Xing Z., Ben-refael A., Amirav L., Paz Y. (2021). Appl. Surf. Sci..

[cit36] Nuber M., Spanier L. V., Roth S., Vayssilov G. N., Kienberger R., Müller-Buschbaum P., Iglev H. (2022). J. Phys. Chem. Lett..

[cit37] Kahmann S., Fazzi D., Matt G. J., Thiel W., Loi M. A., Brabec C. J. (2016). J. Phys. Chem. Lett..

[cit38] Kendrick W. J., Jirásek M., Peeks M. D., Greetham G. M., Sazanovich I. V., Donaldson P. M., Towrie M., Parker A. W., Anderson H. L. (2020). Chem. Sci..

[cit39] Nicolaidou E., Parker A. W., Sazanovich I. V., Towrie M., Hayes S. C. (2023). J. Phys. Chem. Lett..

[cit40] Ovchinnikov O. V., Evtukhova A. V., Kondratenko T. S., Smirnov M. S., Khokhlov V. Y., Erina O. V. (2016). Vib. Spectrosc..

[cit41] Dereka B., Rosspeintner A., Krzeszewski M., Gryko D. T., Vauthey E. (2016). Angew. Chem., Int. Ed..

[cit42] Dereka B., Vauthey E. (2017). J. Phys. Chem. Lett..

[cit43] Szakács Z., Glöcklhofer F., Plasser F., Vauthey E. (2021). Phys. Chem. Chem. Phys..

[cit44] Chen M., Bae Y. J., Mauck C. M., Mandal A., Young R. M., Wasielewski M. R. (2018). J. Am. Chem. Soc..

[cit45] Koch M., Licari G., Vauthey E. (2015). J. Phys. Chem. B.

[cit46] Neelambra A. U., Govind C., Devassia T. T., Somashekharappa G. M., Karunakaran V. (2019). Phys. Chem. Chem. Phys..

[cit47] Coto P. B., Serrano-Andrés L., Gustavsson T., Fujiwara T., Lim E. C. (2011). Phys. Chem. Chem. Phys..

[cit48] Ramakrishna G., Bhaskar A., Goodson T. (2006). J. Phys. Chem. B.

[cit49] Jeong K. S., Pensack R. D., Asbury J. B. (2013). Acc. Chem. Res..

[cit50] Pensack R. D., Asbury J. B. (2011). Chem. Phys. Lett..

[cit51] Stallhofer K., Nuber M., Kienberger R., Körstgens V., Müller-Buschbaum P., Iglev H. (2019). J. Phys. Chem. C.

[cit52] Moser M., Savva A., Thorley K., Paulsen B. D., Hidalgo T. C., Ohayon D., Chen H., Giovannitti A., Marks A., Gasparini N., Wadsworth A., Rivnay J., Inal S., McCulloch I. (2021). Angew. Chem., Int. Ed..

[cit53] Thorley K. J. (2023). J. Phys. Chem. B.

[cit54] Kennehan E. R., Grieco C., Brigeman A. N., Doucette G. S., Rimshaw A., Bisgaier K., Giebink N. C., Asbury J. B. (2017). Phys. Chem. Chem. Phys..

[cit55] Österbacka R., Jiang X. M., An C. P., Horovitz B., Vardeny Z. V. (2002). Phys. Rev. Lett..

[cit56] Minot C., Flytzanis C. (1979). Chem. Phys. Lett..

[cit57] Sachs M., Sprick R. S., Pearce D., Hillman S. A. J., Monti A., Guilbert A. A. Y., Brownbill N. J., Dimitrov S., Shi X., Blanc F., Zwijnenburg M. A., Nelson J., Durrant J. R., Cooper A. I. (2018). Nat. Commun..

[cit58] Balzer D., Kassal I. (2024). Chem. Sci..

[cit59] Balzer D., Kassal I. (2022). Sci. Adv..

[cit60] Sun Z., Jiang Y., Zeng L., Huang L. (2019). ChemSusChem.

[cit61] Chi X., Lan Z. A., Chen Q., Zhang X., Chen X., Zhang G., Wang X. (2023). Angew. Chem., Int. Ed..

