# Supplementary material for: Time-resolved vibrational spectroscopic study of molecular nanoaggregate photocatalysts
Source: Chem Sci. 2024 Sep 13;15(39):16133–41. doi: 10.1039/d4sc03825h (PMC11394368; doi:10.1039/d4sc03825h)
Supplement: SC-015-D4SC03825H-s001 [file SC-015-D4SC03825H-s001.pdf]

Supplementary Information (SI) for Chemical Science.
This journal is © The Royal Society of Chemistry 2024

Supporting information

Time-resolved vibrational spectroscopic study of molecular nanoaggregate photocatalysts

Chao Li,[a] Tao Liu,[b] Owen Thwaites,[c] Adrian M. Gardner,[a] Igor V. Sazanovich,[d] Haofan Yang,[e] Xiaobo Li,[e] Andrew I. Cooper,[b] and Alexander J. Cowan[a]*

[a] Stephenson Institute for Renewable Energy and Department of Chemistry, University of Liverpool, L69 7ZF, UK.

[b] Leverhulme Research Centre for Functional Materials Design, Materials Innovation Factory and Department of Chemistry, University of Liverpool, Liverpool L7 3NY, UK.

[c] Stephenson Institute of Renewable Energy and Department of Physics, University of Liverpool, Liverpool, L69 7ZF, UK.

[d] Central Laser Facility, Research Complex at Harwell, STFC Rutherford Appleton Laboratory, Harwell Campus, Didcot, Oxfordshire OX11 0QX, UK.

[e] Materials Innovation Factory and Department of Chemistry, University of Liverpool, Liverpool, L7 3NY, UK.

Corresponding Author: acowan@liverpool.ac.uk

Table of Contents

1. Experimental Section: 2
  1.1 Synthesis 2
  1.2 Steady state spectroscopy 2
  1.3 Time-Resolved Infrared (TRIR) specterocopy 3
  1.4 Transient absorption (TA) spectroscopy 4
2. Computational methods: 5
3. Supplementary figures 6
4. References 15

1. Experimental Section:

1.1 Synthesis

The synthesis of CNP and nanostructures have been reported in somewhere else.[1] Brifely, 4-(2-Cyanoacetyl)benzonitrile (5 mmol, 0.850 g), 9-phenyl-9*H*-carbazole-3-carboxaldehyde (2.5 mmol, 0.678 g) and ammonium acetate (15 mmol, 1.15 g) were discharged into the reaction flask, then 15 ml acetic acid was added. The reaction mixture was heated to 110 ^{o}C overnight. The resultant precipitates were filtered and washed with methanol. Then, the solids were oxidized with 2,3-dichloro-5,6-dicyano-*p*-benzoquinone (around 5 mmol, 1.13 g) in acetic acid solution (30 ml) under 110 ^{o}C for 1 h, followed by filtration and methanol washing. The solids were purified by column chromatography, giving the final product as an orange powder (around 1.18 g, yield: 83%). Then, the CNP powder was dissolved in anhydrous THF at concentrations of 10 mg ml^{-1} to produce stock solution. 1 ml stock solution was added rapidly into a vial containing 100 ml and 10 ml of DI water under continuous sonication for around 5 s and then removed the THF by placing all samples in a cover-free sample holder on a hot plate at 130 °C for 5 h, resulting in the formation of CNP-s and CNP-f, respectively. The aggregates were dispersed in water (0.1 mg/ml) under continuous sonication for 20 mins and then the aggregate films measured in the TRIR were prepared by 8 drops of aggregates suspension on the surface of 2 mm CaF_2 window following by the evaporation of the water.

1.2 Steady state spectroscopy

UV-Vis spectra were recorded using a Shimadzu UV-2600 spectrophotometer. Attenuated total reflectance-Fourier transfer infrared (ATR-FTIR) spectra of solution and powder were collected on a Bruker Tensor II FTIR spectrometer. ATR spectra were collected for CNP-s and CNP-f powder samples. Prior to the UV-vis measurement CNP solution was prepared in $CHCl_3$ (100μM) , and then transferred into a quartz cuvette, sealed with a rubber septa cap and degassed by Ar bubbling for 10 min. Prior to the FTIR measurement solution sample was

prepared in $CHCl_3$ (100μM) and then transferred in a Harrick Demountable Liquid Cell with a 300 μm Teflon spacer and 2 mm thick CaF_2 windows. The aggregate samples were prepared using the same Harrick cell by sandwiching the CNP aggregate between two CaF_2 windows, in which we use the prepared aggregate film as one window and another is a bare window. All samples were degassed by Ar for about 20 minutes before measurements were taken.

1.3 Time-Resolved Infrared (TRIR) specterocopy

TRIR experiment were carried out by using the LIFEtime laser system at the STFC Rutherford Appleton Laboratory. The time range covered by the setup in a single experiment extends from 200 fs to 1 ms. Details of the experimental apparatus are outlined elsewhere.[2–4] Briefly, 380 nm pump beam (200 nJ per pulse, 1 kHz) was focused to a 150-μm diameter spot on the sample, the cell was continually flowing through the cell. Owing to experiment constraints, the same excitation wavelength, was required for all experiments. We choose 380 nm as it is lower energy than the LE absorption (the two nanoaggregates (Figure 1a) and CNP in solution (Figure S7)), but above energy of the peak of the CT band for all species probed. Two independently tunable, broadband mid-infrared probe beams (≤ 0.05 μJ per pulse, 200 cm^{-1} bandwidths, 100 kHz) were tuned to cover the aromatic ring mode (1400-1600 cm^{-1}) and CN stretching vibration (2100-2280 cm^{-1}) regions. Probe beams were detected by two separate HgCdTe detector arrays after transmission through the sample. Five-second averaging with five repeat cycles was performed for each time delay. A Harrick Demountable Liquid Cell with a 100 μm Teflon spacer and 2 mm thick CaF_2 window was used. CNP solution sample was prepared in $CHCl_3$ (100μM). Aggregetes samples were prepared as drop casting films. Then the cell was degassed by Ar for about 20 minutes before measurements were taken.

Global lifetime analysis (GLA) was carried out within OPTIMUS software. The principles of GLA have been discussed in detail elsewhere.[5–7] GLA wass used to provide a way to visualise complex data sets of time-resolved spectra by decomposing them into a small number of

compartments and to examine the time dependence. Here we used a sequential evolution GLA approach where the combined TRIR data in the region of 1460-1620 and 2110-2280 cm^{-1} is fitted into 3 sequentially excited compartments ($S_1 \rightarrow S_2 \rightarrow S_3 \rightarrow$) and the population of each compartment is modelled by a single exponential decay function, which will be convoluted (⊗) by an instrument response function (IRF(t,λ)), Eq 1. The measured TRIR spectrum consists of the sum of the contributions from each pathway.

$$\Delta A\ (t,\lambda) = \sum_{j=1}^{n} x_j(\tau_j,\lambda)\, e^{-t/\tau_j} \otimes IRF(t,\lambda) \qquad \text{Equation S-1}$$

Where ΔA is the change in optical density, x_j represents the pre-exponential factor, n is the number of compartments, τ_j is the lifetime for each conponent, λ is the wavenumber.

Singular value decomposition (SVD) analysis was carried out on all data sets, and this indicated that three compartments were required in the GLA fitting of TRIR data.

1.4 Transient absorption (TA) spectroscopy

TA spectra (ps–ns timescale) were collected on a Harpia TA spectrometer (Light Conversion) in an experimental configuration reported previously.[1] Brifly the pump light was generated using a Pharos-SP-10W (Light Conversion; ~170 fs, 10 kHz, 1,030 nm) coupled to an OPA (Light Conversion, Orpheus); an internal chopper lowers the pump frequency to 5 kHz. The white-light probe beam was achieved by focusing a portion of the Pharos-SP-10W 1,030 nm output onto a sapphire crystal within the Harpia spectrometer. Samples were excited with a 380 nm pump light with a power of 750 µW. The pump beam (~0.6 mm diameter) and the probe beam (~0.4 mm diameter) were overlapped on the sample position. Data were initially collected using the Harpia TA spectrometer and analysed using Carpetview software (Light Conversion). CNP solution in $CHCl_3$ (100 µM) were prepared and then transferred into a quartz cuvette with a 1 mm path length, sealed with a rubber septa cap and degassed by Ar bubbling for 10 min prior to the measurement.

2. Computational methods:

The ground state structure of CNP was optimized by the Density Functional Theory (DFT) method [8] with CAM-B3LYP functional [9] and Def2-SVP basis set [10] implemented in Gaussian16 [11]. The effects of solvation of $CHCl_3$ was accounted by using the SMD solvation model [12]. The lowest-lying excited state was optimized by Time-dependent density functional theory (TDDFT) method [13] with the same level of theory. The frequency calculations were carried out on the same level and there is no imaginary frequency. The scaling factors are 0.928 in the ring mode and 0.922 in the CN mode, respectively, which were determined such that the scaled wavenumber of the C=C aromatic vibrations and CN stretching vibration modes of CNP matched the experimental ground state wavenumber.

3. Supplementary figures

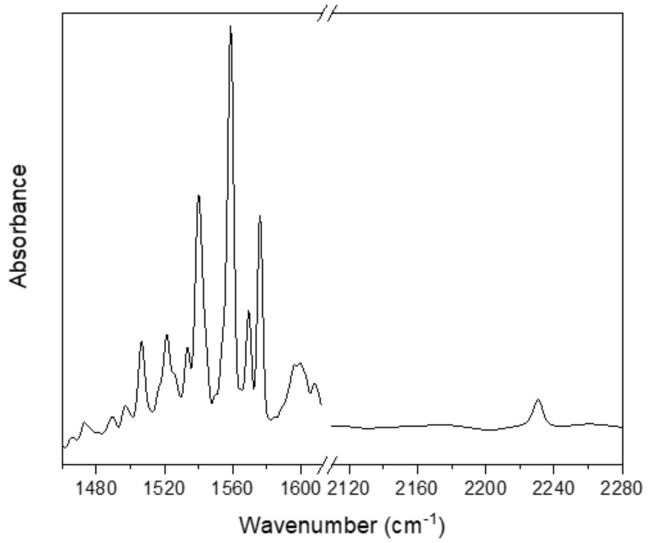

Figure S1. *FTIR spectrum of CNP in solution (CH_3Cl) (100 µM).*

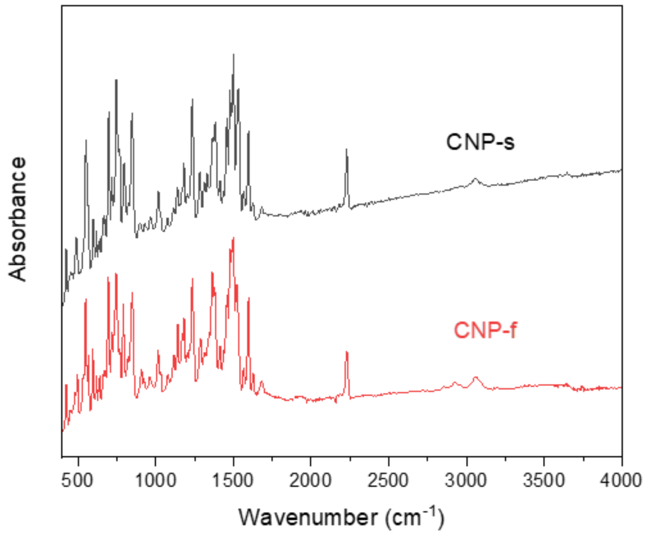

Figure S2. *FTIR-ATR spectra of CNP-s and CNP-f aggregates in solid form (full spectra).*

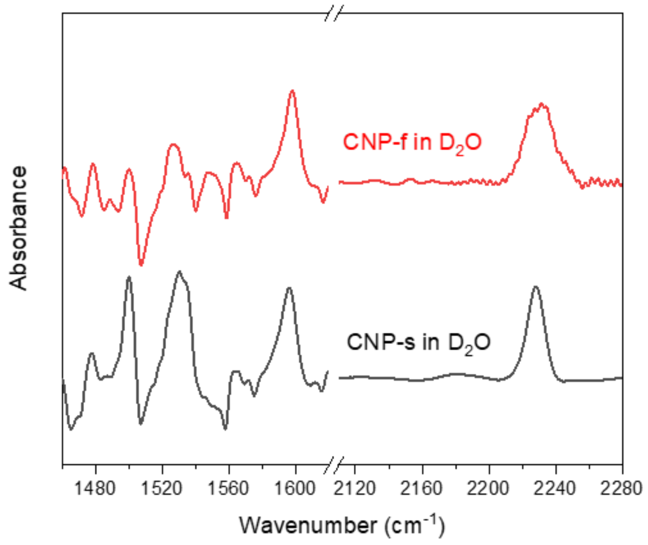

Figure S3. *FTIR spectra of CNP-s and CNP-f in* D_2O *(0.01 mg/mL, PH=7).*

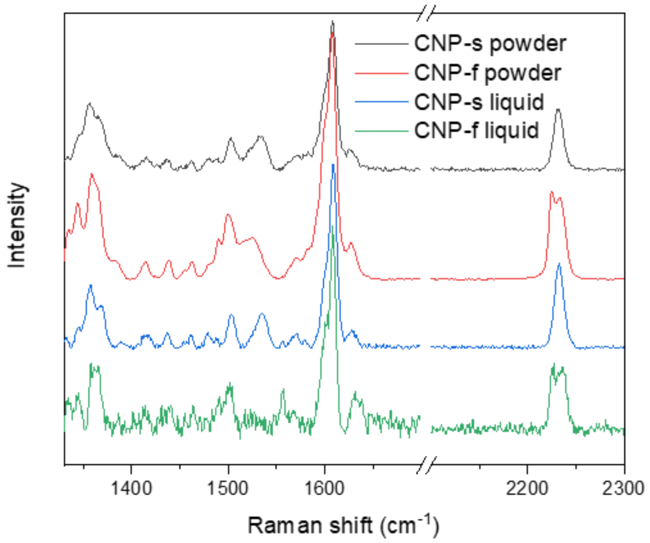

Figure S4. *Ground state Raman spectra (785 nm probe) of CNP-s and CNP-f in water (1 mg/ml) or solid state.*

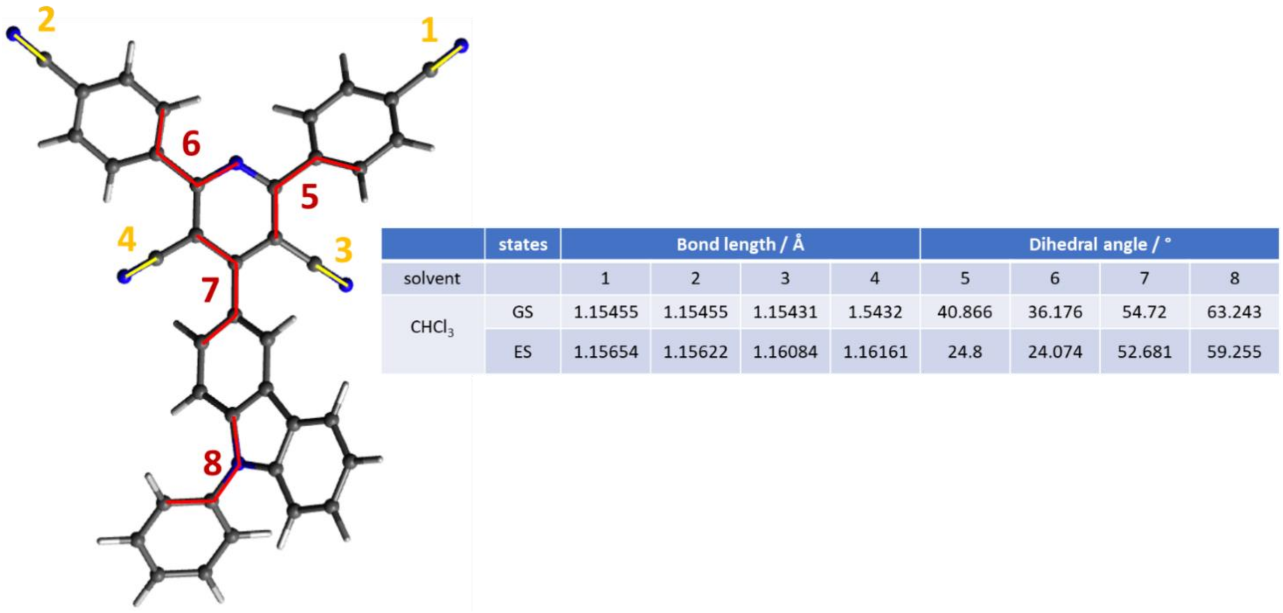

	states	Bond length / Å				Dihedral angle / °			
solvent		1	2	3	4	5	6	7	8
$CHCl_3$	GS	1.15455	1.15455	1.15431	1.5432	40.866	36.176	54.72	63.243
	ES	1.15654	1.15622	1.16084	1.16161	24.8	24.074	52.681	59.255

Figure S5. *Selected bond length and dihedral angles of the ground(GS) and excited state (ES) of CNP in $CHCl_3$ obtained by DFT/TDDFT calculations.*

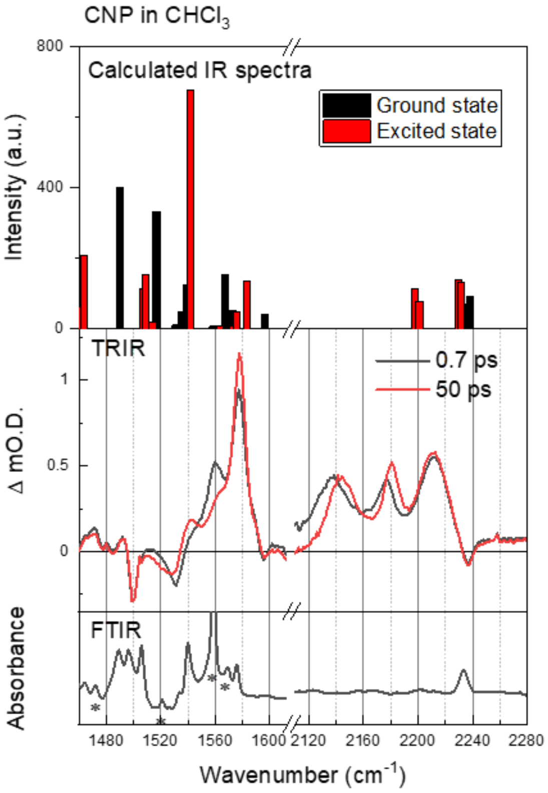

Figure S6. *The comparison of the calculated IR spectra (top panel) of the indicated states, the TRIR spectra probed at 0.7 and 50 ps (middle panel) and the FTIR spectrum (bottom panel) of CNP in $CHCl_3$. The predicted IR spectra were calculated by DFT/TDDFT methods*

implemented in Gaussian16 and the detailed predicted band positions are listed in the Table S1. The asterisks refer to bands from the $CHCl_3$ solvent.

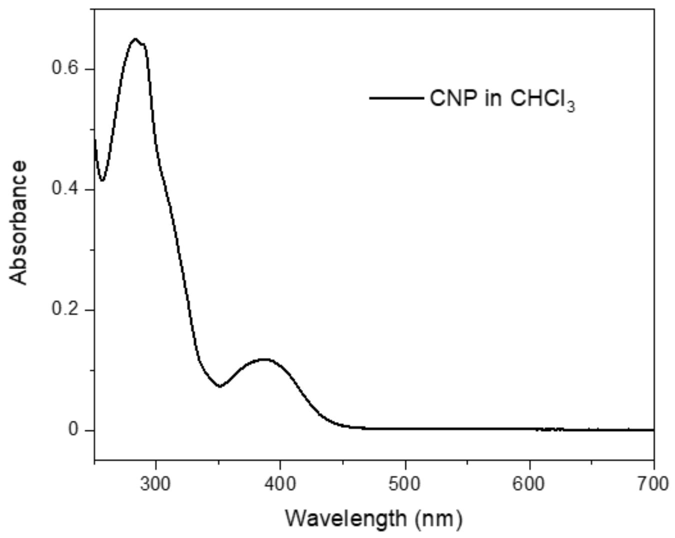

Figure S7. *UV-vis absorption spectrum of CNP in $CHCl_3$ (100 µM) measured in a Harrick demountable liquid cell with a 100 µm Teflon spacer and 2 mm thick CaF_2 window.*

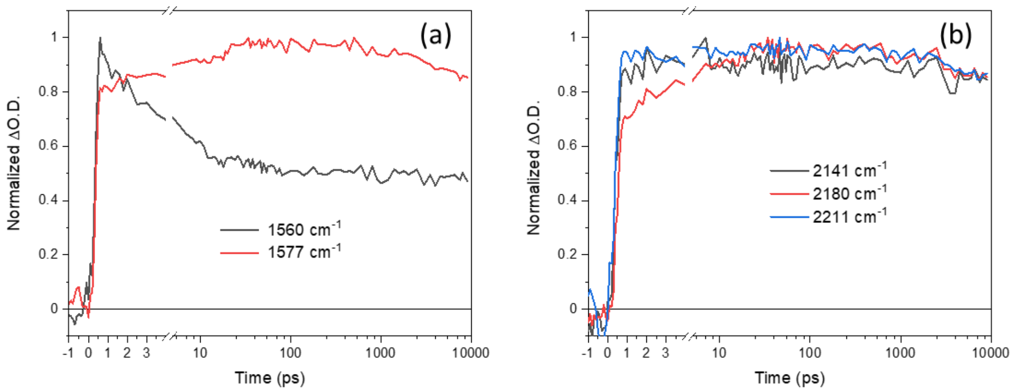

Figure S8. *ps-ns selected normalised kinetic traces of CNP in $CHCl_3$ probed at (a) ring mode, and (b) CN stretching mode.*

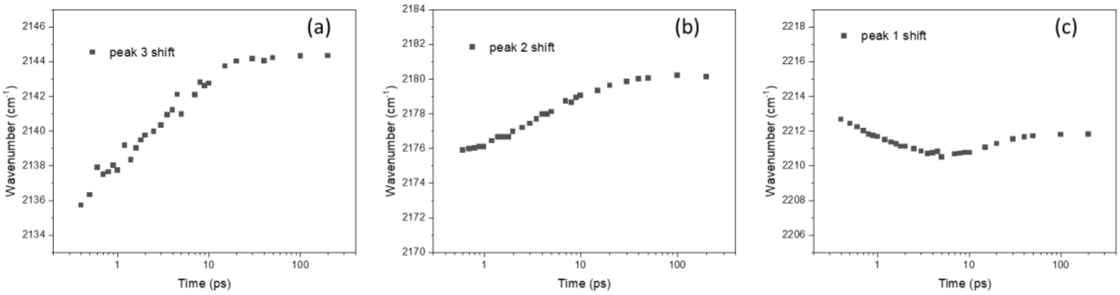

Figure S9. *v(CN) dynamic shifts of peak (a) 3, (b) 2 and (c) 1 (CN vibrations from the lowest to highest frequency) of CNP in $CHCl_3$ obtained from Lorentzian multiple peaks fitting at selected time delays.*

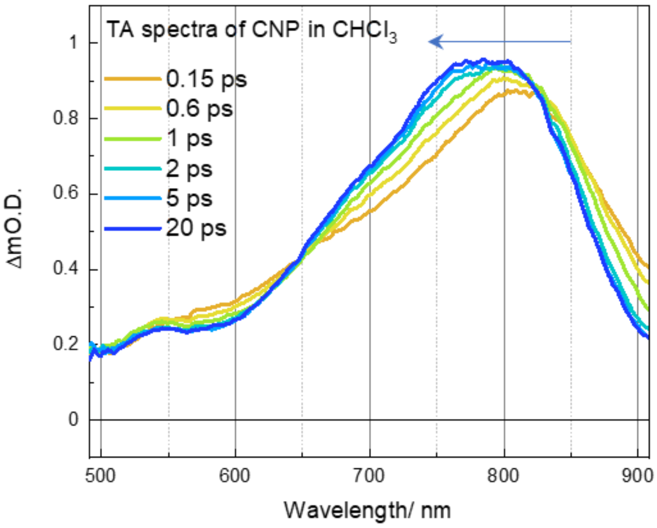

Figure S10. *TA spectra of CNP in $CHCl_3$ (100 µM) following 380 nm excitation, shown at selected time delays. The arrow shows blue-shift of a CT band assigned to reduced acceptor sub-unit.*

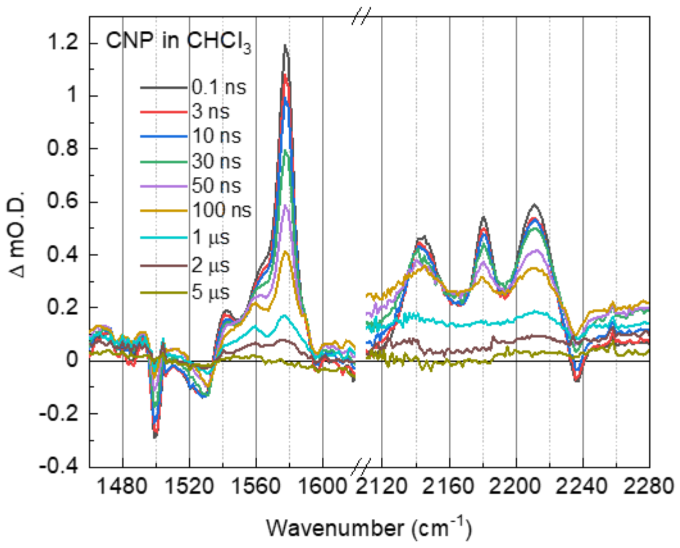

Figure S11. *ns-µs TRIR spectra of CNP in $CHCl_3$ (100µM) following 380 nm excitation at different time delays. It is noted that the broad background signal in the CN mode can be observed >100 ns, which is proposed to be due to potential aggregation.*

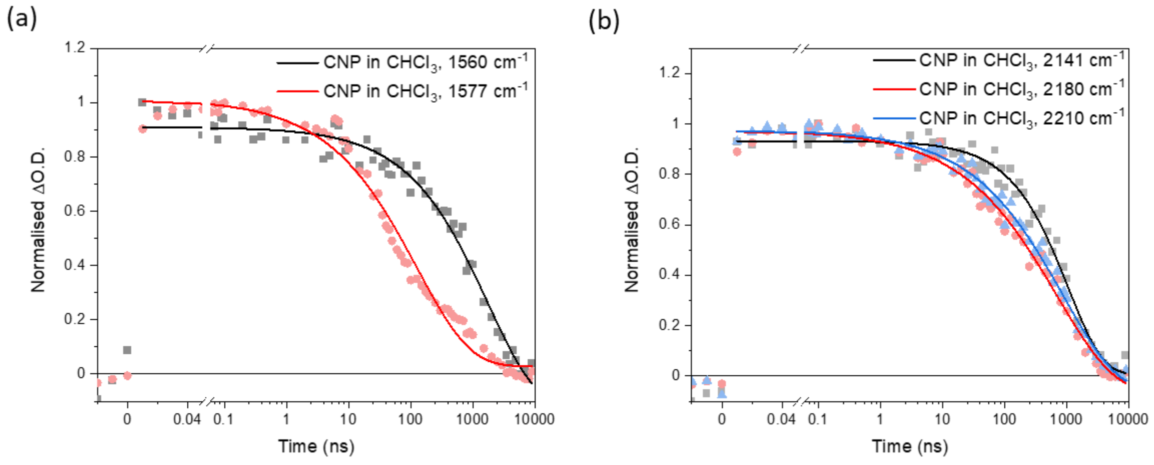

Figure S12. *ns-µs selected normalised kinetic traces of CNP in $CHCl_3$ probed at ring mode (a), and CN stretching mode (b). Single stretched exponential fits are shown (solid dark line, detailed fitting parameters are listed in table S2).*

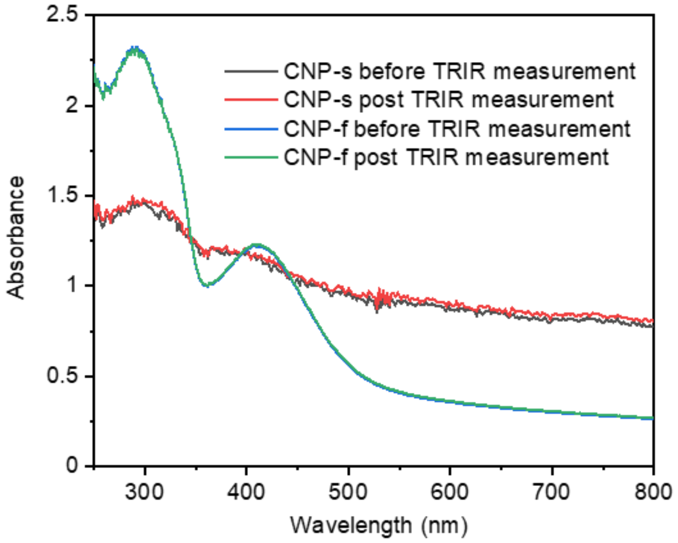

Figure S13. *UV-vis absorption spectra of CNP-s and CNP-f films in transmittance mode recorded before and post TRIR measurement indicated in Figure 3, 4 and 5. Note: the ps-ns and ns-µs TRIR data in the main text are recorded from the same sample (consecutive experiments). The UV-vis absorption spectra are identified before and post two consecutive TRIR measurement, indicating no degradation during the measurement time.*

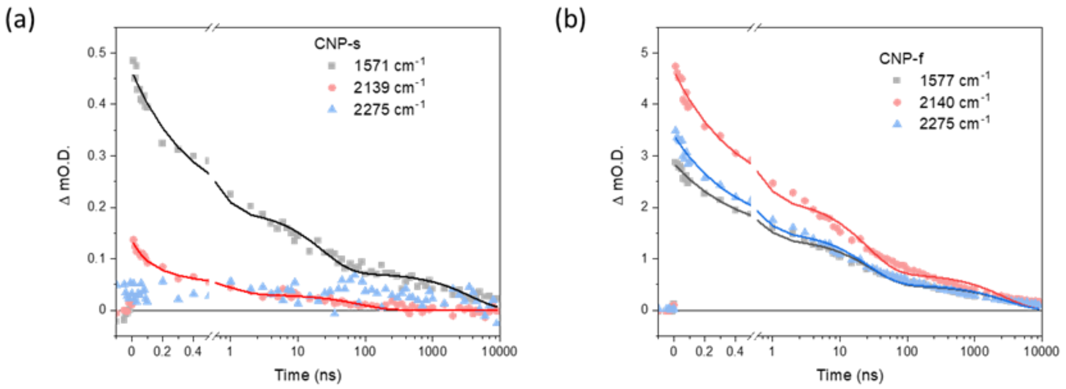

Figure S14. *Kinetic trances of ns-µs TRIR spectra at selected wavenumbers in (a) CNP-s and (b) CNP-f. The resonant TRIR bands (1571 cm^{-1}(CNP-s), 1577 cm^{-1}(CNP-f) in ring mode and 2140 cm^{-1} in CN mode) are chosen, which are overlapped with broad electronic absorption of exitonic state in CNP-s and polaronic state in CNP-f observed in ps-ns TRIR data, respectively. Exponential fits are shown (solid dark line, detailed fitting parameters in table S3 and S4). In CNP-s, the fit can not be obtained at 2275 cm^{-1}due to small amplitude of CT exitonic state and poor signal-to-noise ratio.*

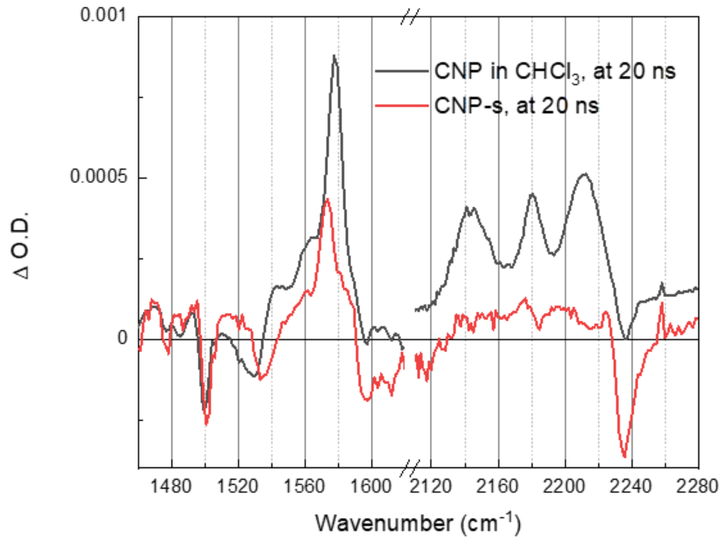

Figure 15. *The comparison of the TRIR spectra of CNP in $CHCl_3$ and CNP-s at 20 ns, showing the spectral similarity of vibrational mode of CNP-s after the decay of the broad background signal with that of localized CT state of CNP in solution.*

Table S1. A tabulated list of calculated vibrations of CNP in $CHCl_3$ by using DFT/TDDFT methods implemented in Gaussian 16. Calculated wavenumbers are scaled with scaling factors of 0.9277 and 0.925 for ring mode and CN mode, respectively

CNP in $CHCl_3$				*Characterization*
Ground state		*Excited state*		
wavenumber (cm^{-1})	*intensity (a.u.)*	*wavenumber (cm^{-1})*	*intensity (a.u.)*	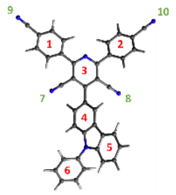
1456.2	*58.8*	*1457.0*	*63.9*	*carbon backbone of 1,2*
1492.2	*402.4*	*1461.5*	*210.4*	*carbon backbone of 4,5,6,*
1519.3	*333.9*	*1504.3*	*113.2*	*carbon backbone of 1,2,4*
1533.5	*12.2*	*1506.7*	*154.9*	*carbon backbone of 4*
1537.6	*50.8*	*1511.5*	*20.7*	*carbon backbone of 1,2*
1541.1	*126.2*	*1539.1*	*678.6*	*carbon backbone of 3*
1548.8	*0.5*	*1560.8*	*9.2*	*carbon backbone of 6*

1560.7	*7.9*	*1570.9*	*8.4*	*carbon backbone of 5*
1569.7	*155.8*	*1573.3*	*50.6*	*carbon backbone of 6*
1575.0	*53.5*	*1580.8*	*135.0*	*carbon backbone of 4,5*
1586.1	*0.2*	*1586.5*	*0.2*	*carbon backbone of 1*
1586.6	*0.02*	*1587.7*	*0.2*	*carbon backbone of 2*
1598.5	*41.6*	*1614.0*	*234.0*	*carbon backbone of 4*
2237.4	*70.9*	*2194.9*	*115.5*	*CN stretching vibration of 7*
2238.1	*32.3*	*2198.4*	*79.2*	*CN stretching vibration of 8*
2240.4	*91.7*	*2227.1*	*138.4*	*CN stretching vibration of 10*
2240.7	*73.5*	*2228.7*	*132.2*	*CN stretching vibration of 9*

Table S2. Single stretched exponential fits ($y = A * e^{-\left(\frac{x}{\tau}\right)^{\beta}}$) result of the dynamics of ns-μs TRIR spectra of CNP in $CHCl_3$ at selected wavenumbers.

Wavenumber (cm^{-1})	*1560*	*1577*	*2141*	*2180*	*2210*
τ (ns)	*1750 ± 391*	*127 ± 11*	*1069 ± 89*	*702± 91*	*880 ± 100*
β	*0.57 ± 0.04*	*0.51 ± 0.03*	*0.78 ± 0.046*	*0.47 ± 0.02*	*0.49 ± 0.02*
A	*1.02 ± 0.07*	*0.98 ± 0.02*	*0.92 ± 0.02*	*1.04 ± 0.03*	*1.04 ± 0.03*

Table S3. Exponential fits result of the dynamics of ns-μs TRIR spectra of CNP-s at selected wavenumbers.

Wavenumber (cm^{-1})	*1571*	*2140*
τ_1 (ns)	*0.39 ± 0.036*	*0.10 ± 0.048*
A_1	*$2.7\times10^{-4} \pm 8.9\times10^{-6}$*	*$5.5\times10^{-5} \pm 1.8\times10^{-5}$*

τ_2 (ns)	23.16 ± 3.76	0.76 ± 0.40
A_2	$1.2\times10^{-4} \pm 7.7\times10^{-6}$	$5.1\times10^{-5} \pm 2\times10^{-5}$
τ_3(ns)	3747.55 ± 691.33	84.15 ± 17.21
A_3	$7.2\times10^{-5} \pm 4\times10^{-6}$	$3.1\times10^{-5} \pm 2.4\times10^{-6}$
AWL (ns)	590 ± 136	19.4 ± 1.2

Table S4. Exponential fits result of the dynamics of ns-µs TRIR spectra of CNP-f at selected wavenumbers.

Wavenumber (cm^{-1})	1577	2140	2275
τ_1 (ns)	0.44 ± 0.037	0.40 ± 0.033	0.40 ± 0.032
A_1	$1.46\times10^{-3} \pm 4.1\times10^{-5}$	$2.5\times10^{-3} \pm 7.2\times10^{-5}$	$1.9\times10^{-3} \pm 5.2\times10^{-5}$
τ_2 (ns)	29.39 ± 3.08	25.44 ± 2.89	26.03 ± 3.03
A_2	$9.1\times10^{-4} \pm 3.6\times10^{-5}$	$1.5\times10^{-3} \pm 6.2\times10^{-5}$	$1.0\times10^{-3} \pm 4.5\times10^{-5}$
τ_3(ns)	3056.66 ± 439.15	2725.63 ± 445.53	2943.97 ± 495.14
A_3	$4.8\times10^{-4} \pm 2.2\times10^{-5}$	$7\times10^{-4} \pm 3.7\times10^{-5}$	$5\times10^{-4} \pm 2.7\times10^{-5}$
AWL (ns)	523 ± 98	414 ± 71	444 ± 108

4. References

1 H. Yang, C. Li, T. Liu, T. Fellowes, S. Y. Chong, L. Catalano, M. Bahri, W. Zhang, Y. Xu, L. Liu, W. Zhao, A. M. Gardner, R. Clowes, N. D. Browning, X. Li, A. J. Cowan and A. I. Cooper, *Nat Nanotechnol*, 2023, **18**, 307–315.

2 W. J. Kendrick, M. Jirásek, M. D. Peeks, G. M. Greetham, I. V. Sazanovich, P. M. Donaldson, M. Towrie, A. W. Parker and H. L. Anderson, *Chem Sci*, 2020, **11**, 2112–2120.

3 G. M. Greetham, P. Burgos, C. Qian, I. P. Clark, P. S. Codd, R. C. Farrow, M. W. George, M. Kogimtzis, P. Matousek, A. W. Parker, M. R. Pollard, D. A. Robinson, X. Zhi-Jun and M. Towrie, *Appl Spectrosc*, 2010, **64**, 1311–1319.

4 L. Lewis-Borrell, M. Sneha, I. P. Clark, V. Fasano, A. Noble, V. K. Aggarwal and A. J. Orr-Ewing, *J Am Chem Soc*, 2021, **143**, 17191–17199.

5 Y. Bai, C. Li, L. Liu, Y. Yamaguchi, M. Bahri, H. Yang, A. Gardner, M. A. Zwijnenburg, N. D. Browning, A. J. Cowan, A. Kudo, A. I. Cooper and R. S. Sprick, *Angew Chem Int Ed*, 2022, **61**, e202201299.

6 C. Slavov, H. Hartmann and J. Wachtveitl, *Anal Chem*, 2015, **87**, 2328–2336.

7 I. H. M. van Stokkum, D. S. Larsen and R. van Grondelle, *Biochim Biophys Acta*, 2004, **1657**, 82–104.

8 W. Kohn, A. D. Becke and R. G. Parr, *J Phys Chem*, 1996, **100**, 12974–12980.

9 T. Yanai, D. P. Tew and N. C. Handy, *Chem Phys Lett*, 2004, **393**, 51–57.

10 F. Weigend and R. Ahlrichs, *Phys Chem Chem Phys*, 2005, **7**, 3297.

11 M. J. Frisch, G. W. Trucks, H. B. Schlegel, G. E. Scuseria, M. a. Robb, J. R. Cheeseman, G. Scalmani, V. Barone, G. a. Petersson, H. Nakatsuji, X. Li, M. Caricato, a. V. Marenich, J. Bloino, B. G. Janesko, R. Gomperts, B. Mennucci, H. P. Hratchian, J. V. Ortiz, a. F. Izmaylov, J. L. Sonnenberg, Williams, F. Ding, F. Lipparini, F. Egidi, J. Goings, B. Peng, A. Petrone, T. Henderson, D. Ranasinghe, V. G. Zakrzewski, J. Gao, N. Rega, G. Zheng, W. Liang, M. Hada, M. Ehara, K. Toyota, R. Fukuda, J. Hasegawa, M. Ishida, T. Nakajima, Y. Honda, O. Kitao, H. Nakai, T. Vreven, K. Throssell, J. a. Montgomery Jr., J. E. Peralta, F. Ogliaro, M. J. Bearpark, J. J. Heyd, E. N. Brothers, K. N. Kudin, V. N. Staroverov, T. a. Keith, R. Kobayashi, J. Normand, K. Raghavachari, a. P. Rendell, J. C. Burant, S. S. Iyengar, J. Tomasi, M. Cossi, J. M. Millam, M. Klene, C. Adamo, R. Cammi, J. W. Ochterski, R. L. Martin, K. Morokuma, O. Farkas, J. B. Foresman and D. J. Fox, 2016, Gaussian 16, Revision C.01, Gaussian, Inc., Wallin.

12 A. V. Marenich, C. J. Cramer and D. G. Truhlar, *J Phys Chem B*, 2009, **113**, 6378–6396.

13 Q. Wu and T. Van Voorhis, *Phys Rev A*, 2005, **72**, 7–10.
